# Sanitary Reform

**Published:** 1850-04

**Authors:** 


### SANITARY REFORM.

The devastations of cholera in England have been useful, in turning the attention of men to the causes of disease, and to the means of re moving them. We should be pleased to see a similar spirit of investigation and improvement aroused in this country, and we earnestly call the attention of our medical brethren to the subject. There are few objects on which they can more profitably spend their exertions, and there are none likely to be fraught with more useful results. The labors of the sanitary boards in England have shown a most frightful condition in the habitations of the poor, and in municipal regulations. In surveying this development, instead of feeling surprize at the extent of the ravages of typhus and typhoid fever, and of cholera, in particular localities, we are only surprised that any one, in the infected districts, should have survived. But while contemplating the baleful influences that ravage the lives of men, there is consolation in the fact, that preventive services can be efficiently rendered. These are the services which humanity has a right to demand of physicians, and to the honor of our profession, it should be said that its members have shown themselves ready at all times to remove the causes of disease.

The Edinburgh Review, for January, 1850, says: "In England alone, the average annual number of deaths from disease is, in round numbers, 300,000,—while that of deaths from the mere decay and exhaustion of the human frame, by the progress of time, is only 35,000. In the difference between these two numbers we see the vast and vital field in which the sanitary reformer proposes to work. That disease shall ever be entirely exterminated, is of course beyond the belief or hope of the most sanguine: but every disease has somewhere its specific and efficient cause—and that these causes can be removed, or much weakened in their action, in very many instances, is not only within the bounds of hope, but has been satisfactorily proved."

In reference to sanitary improvements, in vogue at present, the Edinburgh Review says:

"Their object is not the vain one of indefinitely protracting existence by human art. It seems, indeed, a remarkable fact, whence something has yet, we think, to be learned, that wherever the average length o[illegible] life is improved, there is a tendency to equality; and that the miraculously long livers appear chiefly where the average vitality is shortest, and where men subject to noxious agencies present in general the most at tenuated and degraded physical condition,—as if it were an exceptional law of nature, that those whose frames can, for a certain time, resist these deadly influences, become, 'like him who fed on poisons,' hardened against the common causes of mortality. Nor, were we to suppose it in the power of art materially to enlarge the allotted span of healthy existence, and to make extreme old age the common lot, instead of the rare privilege of a few,—must we necessarily suppose the boon accompanied by a great increase of human happiness. Treatises *de senectute* have more than one aspect. In the general case, when a green old age has been reached, the task of life may be considered to have been performed, and its enjoyments substantially exhausted; the journey has been completed, and the tired body may part with the spirit in peace, leaving the arena of life to new competitors, The associations with which survivors surround the memory of their aged relatives are accordingly more tender than bitter. And such deaths, it is felt, do not so much make a chasm in existence as a natural change, consonant with the decay and reproduction which are the routine of the world's progress. Young hands are strengthening their grasp to lead us forward in our journey through life, as the elder race waxes feeble and drops away. It is when the opening bud is blighted, or life is cut off in the full bloom of usefulness,—in the midst of happiness, affection, and esteem,—that the great calamities of mortality are exhibited. Such are the desolate spots of human existence, standing in the centre of its healthy fruition, waste and arid—showing happy aims defeated, and its joys engulphed in unfathomable sorrows. The science that promises in some measure to mitigate the horrors of this howling wilderness, is surely an object before which sarcasm and faction and selfishness may well be dumb."

These are certainly high and ennobling considerations, and if they are not worthy the attention of medical men, we should be at a loss to imagine anything that can be.

A national association of physicians has been formed in this country, which bids fair to be productive of a large amount of good. At the last meeting of this association, a large amount of information on sanitary subjects was gathered, and we hope that there will be an annual increase of valuable knowledge on these important points.

